# Distinct IgE sensitization profiles in chronic urticaria: a comparative study with classic allergic diseases

**DOI:** 10.3389/fimmu.2024.1458839

**Published:** 2024-12-05

**Authors:** Xianjie Yang, Shifei Li, Anqi Chen, Huan Wang, Sisi Deng, Bing Ni, Zhiqiang Song, Qiquan Chen

**Affiliations:** ^1^ Department of Dermatology, Southwest Hospital, Army Medical University, Chongqing, China; ^2^ Department of Pathophysiology, Army Medical University, Chongqing, China

**Keywords:** chronic urticaria, atopy, IgE sensitization, allergen, allergic disease

## Abstract

**Introduction:**

Chronic urticaria (CU) is not traditionally classified as an allergic disease, but emerging evidence suggests a link to atopy. The quintessential marker of atopy is IgE sensitization, there is scarce information on the IgE sensitization characteristics of CU.

**Methods:**

To investigate IgE sensitization characteristics in CU, and compare them with classic allergic diseases. We retrospectively analyzed the results of specific IgE (sIgE) and total IgE (tIgE) in CU patients, explored the distribution patterns of these atopic markers in CU, and compared these data with those of atopic dermatitis (AD), allergic rhinitis (AR), asthma (AS), and healthy controls (HC).

**Results:**

1149 patients (396 CU, 411 AD, 101 AR, 139 AS and 102 HC) were included in the study. 33.1% of CU patients showed positive sIgE and 49.0 % had elevated tIgE levels, significantly higher than those in HC. Comparative analysis with classic allergic diseases showed CU patients had a lower sIgE positivity rate but no significant difference in tIgE levels. Gender and age influenced sensitization profiles, with male CU patients showing a higher sIgE positivity rate. The distribution of sIgE levels, allergen categories, and tIgE elevated levels range in CU differed from classic allergic disease. The concordance rate between sIgE and tIgE results in CU was lower than in classic allergic disease.

**Conclusion:**

Our study reveals that a significant proportion of CU patients display IgE sensitization, suggesting a clear atopic background compared to the general population. However, the IgE sensitization profile in CU differs from that of classical allergic diseases such as AD, AR, and AS, characterized by relatively lower intensity of IgE sensitization. The underlying reasons for this phenomenon and its clinical implications in CU warrant further research.

## 1 Introduction

Chronic urticaria (CU) stands as one of the most prevalent chronic conditions within the field of dermatology, affecting up to 2.6% of the Chinese population (1). It significantly diminishes the quality of life and work productivity of those afflicted, imposing a substantial burden on society at large (2). The etiology of CU remains largely enigmatic, with autoimmunity, coagulation cascades, vitamin D deficiency, infections, and emotional factors all implicated in its development (3). Yet, for over half of CU patients, the known mechanisms fail to fully account for their condition.

Atopy is characterized by a genetic predisposition to produce IgE antibodies and become sensitized to commonly encountered allergens (4). The quintessential marker of atopy is IgE sensitization, which manifests clinically as positive allergen-specific IgE (sIgE) tests and elevated serum total IgE (tIgE) levels. While urticaria is frequently cited as a quintessential cutaneous manifestation of immediate-type allergic reactions, CU symptoms often do not correlate with exposure to specific allergens. The latest international guidelines do not incorporate allergen testing as a mandatory examination for CU patients (5). As a result, researchers frequently neglect the potential role of allergens or atopic backgrounds in CU, even though this is a matter of frequent concern among patients in clinical practice. Nonetheless, an emerging body of clinical evidence indicates that a significant proportion of CU patients possess an atopic background (6–10). Moreover, in recent years, monoclonal antibodies directed against free IgE, such as omalizumab, have demonstrated promising therapeutic effects for CU (11). Some researchers have observed a correlation between the therapeutic response to omalizumab and the atopic status of CU patients, albeit with varying outcomes across different studies (12–14). Collectively, these findings indicate the intricate and close link between CU and atopy. However, research on the connection between atopy and CU is still sparse, particularly regarding the IgE sensitization profile of CU patients. The distinctions between the IgE sensitization characteristics of CU and those of classical atopic diseases are not yet well-defined.

This study is designed to preliminary analysis the IgE sensitization characteristics of CU patients, utilizing the findings from allergen-sIgE testing and serum tIgE levels. We conducted a thorough and in-depth analysis of the sensitization profiles of CU and classical allergic diseases, such as atopic dermatitis (AD), allergic rhinitis (AR), asthma (AS). The insights gained from this study will enhance our comprehension of the association between CU and atopy, potentially leading to more targeted and effective treatment strategies.

## 2 Methods

### 2.1 Study design and subject enrollment

All patients who underwent both sIgE and serum tIgE tests at the Urticaria Center of Reference and Excellence (UCARE) (15) of the Southwest Hospital (Chongqing, China) from January 2023 to December 2023 were included and retrospectively analyzed. The diagnose of the patients were by specialists according to the corresponding diagnostic criteria, such as international EAACI/GA²LEN/EuroGuiDerm/APAAACI guideline for CU (5), Chinese Criteria for AD (16), Chinese Society of Allergy Guidelines for diagnosis of AR (17), and Chinese guidelines for the diagnosis of AS (18). Cases with an unknown or suspicious diagnosis were excluded, and patients whose final diagnosis was not CU or any of the above classical allergic diseases were excluded. Patients with more than one coexisting condition were included only according to the leading diagnosis. The leading diagnosis is ascertained based on the condition that most significantly affects the patient’s quality of life and for which the patient was primarily seeking medical attention. Furthermore, any comorbidities derived from the patient’s past medical history are not considered as the leading diagnosis. Each subject has only one leading diagnosis, and there are no instances of the same patient being repeatedly included in the study. Additionally, all subjects with classic allergic diseases included in the study did not have comorbid CU. In addition, healthy individuals, who were not diagnosed with urticaria or allergic diseases and had no history of allergic diseases, underwent testing for tIgE and sIgE as part of their routine health examination during the same time interval were enrolled as the healthy control group.

### 2.2 Detection of sIgE and tIgE

Both tIgE and sIgE tests were performed by the Clinical Laboratory Department of Southwest Hospital, Army Medical University. The laboratory follows Good Clinical Laboratory Practice (GCLP) standards to ensure the accuracy and reliability of the test results. tIgE assays were performed using a fully automated specific protein analysis system (IMMAGE 800, Beckman Coulter, Inc., USA), and human serum total IgE levels were quantified by rate scattering turbidimetry. A normal level of tIgE is <100 IU/ml, while ≥100 IU/ml is considered positive or elevated. tIgE levels of 100-499 IU/ml, 500-999 IU/ml, and≥1000 IU/ml were regarded as slightly elevated, moderately elevated, and highly elevated (19). sIgE was detected by the AllergyScreen allergen detection system (Mediwiss Analytic, Germany), which semi-quantitatively detected human serum allergen-sIgE antibody content by immunoblotting. A total of 29 common allergens were detected, including 18 aeroallergens allergens and 11 food allergens. Referring to the previous study (20), we divided the aeroallergens into five subgroups and the food allergens into two subgroups, as shown in **Supplementary Table S1**. Based on the level of sIgE, the results of each allergen-sIgE were graded as follows: Grade 0 was defined as <0.35 IU/ml, grade 1 as 0.35-0.69 IU/ml, grade 2 as 0.70-3.49 IU/ml, grade 3 as 3.5-17.49IU/ml, grade 4 as 17.5-49.9 IU/ml, grade 5 as 50-100 IU/ml, and grade 6 as >100 IU/ml. Positive sIgE is defined as a positive result in one or more allergen sIgE tests, otherwise it was negative. Positive sIgE levels were divided into low level (grade 1 to 2), medium level (grade 3 to 4) and high level (grade 5 to 6), and positive sIgE numbers were divided into single sensitization (1), some sensitization (2 to 4) and multiple sensitization (5 or more) (21).

### 2.3 Statistics

Data statistical analysis by SPSS 26.0 software. Shapiro-Wilk was used to test the normality of quantitative data. Data that were normally distributed were described using the mean ± standard deviation (X ± SD), while data that were not normally distributed were described using the median and interquartile range (M (25, 75)). Results for categorical data were expressed as counts (n) and percentages (%). For comparisons between two groups of non-normally distributed quantitative data, the Mann-Whitney U test was used, and for comparisons among multiple groups, the non-parametric Kruskal-Wallis test was applied. Chi-square test was used to analyze the differences in the distribution of categorical variables across groups. Statistical significance was determined at a p-value of less than 0.05. *Post-hoc* pairwise comparisons were adjusted using the Bonferroni method.

## 3 Results

### 3.1 Patient characteristics

From January to December 2023, a total of 2,139 patients at the Southwest Hospital of the Army Medical University underwent simultaneous serum sIgE and tIgE testing. According to the diagnostic criteria, 1149 cases were diagnosed by the corresponding specialists and included in this study, comprising 396 cases of CU, 411 cases of AD, 101 cases of AR, 139 cases of AS, and 102 healthy controls (HC). There were 492 male patients (42.8%) and 657 female patients (57.2%). The median age (IQR) was 29 (14, 46) years old, with 233 cases (20.3%) aged 0 to 11 years, 130 cases (11.3%) aged 12 to 17 years, 706 cases (61.4%) aged 18 to 59 years, and 80 cases (7.0%) aged 60 years or older.

### 3.2 CU patients have a higher positive rate of sIgE and higher levels of tIgE than healthy individuals

We conducted a comparative analysis between CU and HC, finding no statistical differences in age and gender between the two groups. In terms of IgE sensitization characteristics, the positive rate of sIgE in CU patients reached 33.1%, which is significantly higher than the allergen positivity rate in the HC (16.7%). Specifically, the positive rates for inhalant and food allergens in CU patients were 31.1% and 6.6%, respectively, both significantly higher than those in the healthy group (15.7% and 1.0%, respectively). 49.0% of CU patients had elevated levels of tIgE, compared to 19.6% HC. The median tIgE level (IQR) in CU patients was 96.7 (43.1, 193.0) IU/ml, also significantly higher than that in the healthy group, which was 32.0 (16.7, 86.2) IU/ml (**Table 1**).

**Table 1:** Comparison of demographic and atopic characteristics between CU and healthy individuals.

	CU	HC	*P*
Sex, M:F	146:250	28:74	0.075^a^
Age (y), M (IQR)	35 (24, 48)	31 (16, 47)	0.081^b^
sIgE+, n (%)	131 (33.1)	17 (16.7)	0.001^a^
aeroallergens	123 (31.1)	16 (15.7)	0.002^a^
food allergens	26 (6.6)	1 (1.0)	0.026^a^
tIgE+, n (%)	194 (49.0)	20 (19.6)	<0.001^a^
tIgE, M (IQR), IU/mL	96.7 (43.1, 193.0)	32.0 (16.7, 86.2)	<0.001^b^

M: F, Male: Female; M (IQR), Median (interquartile range); CU, chronic urticaria; HC, healthy controls.

a Pearson chi-squared test.

b Mann-Whitney U test.

### 3.3 The positive rate of sIgE in CU patients was lower than that in classical allergic diseases, but the level of tIgE was not

We compared the IgE sensitization characteristics of CU with those of classic allergic diseases such as AD, AR and AS. The results showed that the positive rate of sIgE in CU (33.1%) was significantly lower than in AD (51.8%), AR (64.4%) and AS (48.2%) (all statistically significant), with this characteristic being particularly pronounced for inhalant allergens, while only the food allergen positivity rate in AD was significantly higher than in CU. There was no significant difference in the proportion of patients with elevated tIgE levels between CU and classic allergic diseases. Although the tIgE levels in CU patients were significantly lower than in AD, there was no significant difference compared to AR and AS (**Table 2**).

**Table 2:** Comparison of sIgE and tIgE characteristics between patients with CU and classical allergic diseases.

	CU	AD	AR	AS	*P*
sIgE+, n (%)	131 (33.1)	213 (51.8)*	65 (64.4)*	67 (48.2)*	<0.001^a^
aeroallergens	123 (31.1)	189 (46.0)*	63 (62.4)*	63 (45.3)*	<0.001^a^
food allergens	26 (6.6)	65 (15.8)*	13 (12.9)	10 (7.2)	<0.001^a^
tIgE+, n (%)	194 (49.0)	220 (53.5)	55 (54.5)	64 (46.0)	0.316^a^
tIgE, M (IQR), IU/mL	96.7 (43.1, 193.0)	114.0 (38.4, 524.0)*	109.0 (36.0, 242.0)	83.6 (38.1, 209.5)	0.009^b^

IQR, interquartile range; CU, chronic urticaria; AD, atopic dermatitis; AR, allergic rhinitis; AS, asthma.

a Pearson chi-squared test.

b Kruskal-Wallis test.

*Bonferroni correction was used for *post-hoc* analysis. Compared with the CU group, P<0.0167 (0.05/3) was considered statistically significant.

### 3.4 The distribution of sIgE and tIgE in CU was different from that in classical allergic diseases by age and gender

We further analyzed the impact of gender and age on the IgE sensitization characteristics of the diseases. For sIgE, only male CU patients had a higher positive rate of sIgE than female CU patients (P=0.032), while there was no statistical difference in the positive rate of sIgE between genders in AD, AR, and AS patients (**Supplementary Figure S1A**). In terms of age, only in CU, there was no significant difference in the positive rate of sIgE between minors and adults, while the positive rate of sIgE in minors was significantly higher than in adults for AD, AR, and AS (**Supplementary Figure S1B**). For tIgE, CU, like AD and AS, had significantly higher tIgE levels in males than in females, with only AR showing no statistical difference in tIgE levels between genders. In terms of age, there was no significant difference in tIgE levels between minors and adult patients in CU and AS, while the tIgE levels in minors were significantly higher than in adult patients for AD and AR (**Table 3**).

**Table 3:** Comparison of tIgE levels between sex and age.

	CU	AD	AR	AS
Sex, tIgE, M (IQR), IU/mL				
Male	115.0 (54.9, 212.0)	175.5 (42.0, 607.0)	148.0 (51.2, 334.0)	131.0 (49.1, 337.0)
Female	90.8 (39.5, 168.0)	89.4 (34.1, 384.5)	91.8 (25.2, 213.0)	69.1 (31.9, 168.0)
*P* ^a^	0.006	0.008	0.062	0.021
Age, tIgE, M (IQR), IU/mL				
Minors^b^	112.0 (43.9, 198.5)	134.0 (48.1, 689.0)	181.0 (72.6, 567.0)	170.0 (38.2, 908.0)
Adults	96.1 (43.5, 179.0)	92.7 (31.0, 440.0)	89.5 (29.5, 185.0)	77.7 (37.9, 196.0)
*P* ^a^	0.834	0.007	0.002	0.351

IQR, interquartile range; CU, chronic urticaria; AD, atopic dermatitis; AR, allergic rhinitis; AS, asthma.

^a^Mann-Whitney U test. ^b^Minors, including both the 0-11 and 12-17 age groups.

### 3.5 CU shows a lower degree of IgE sensitization than classical allergic diseases

**Figure 1:**
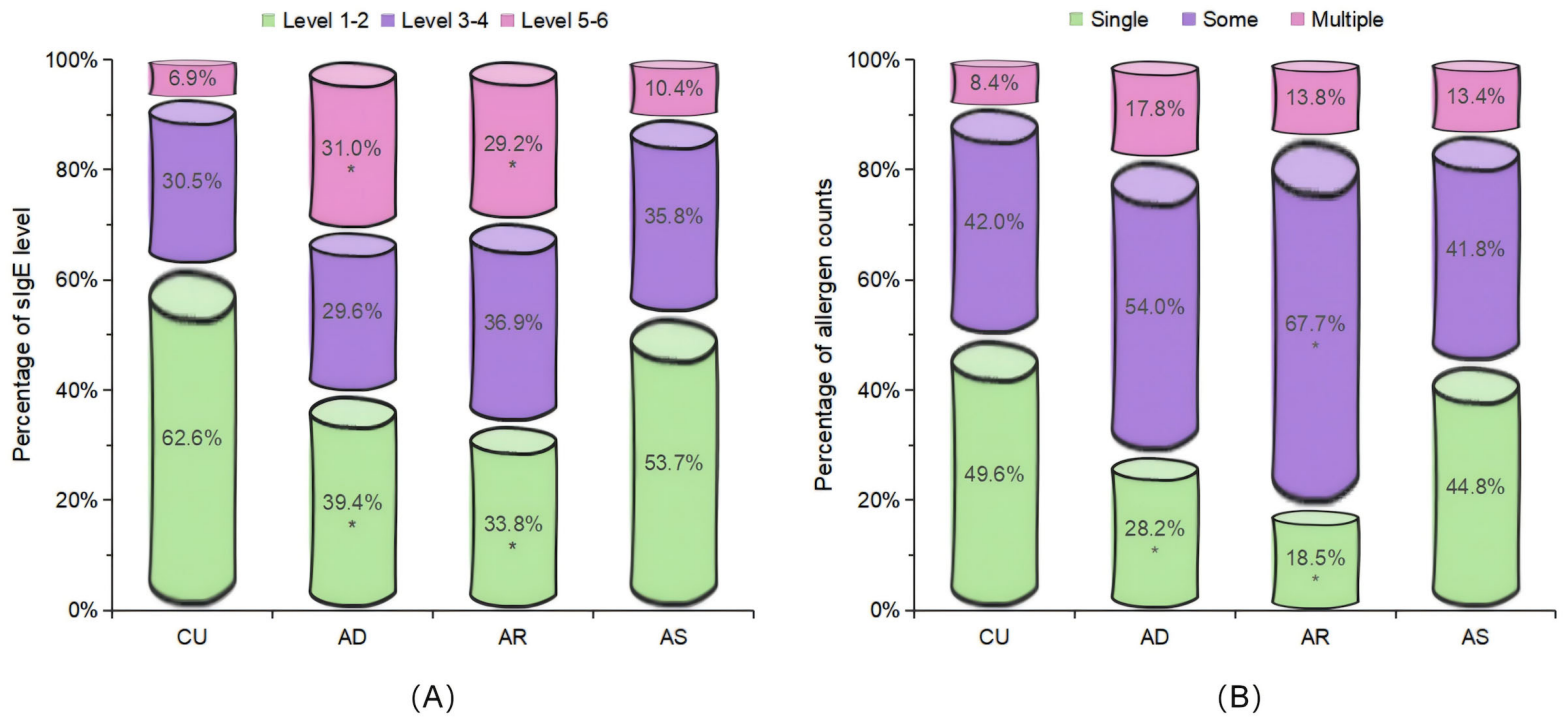
Comparison of the distribution characteristics of the sIgE levels and the positive allergen counts. Included were patients with positive sIgE. **(A)** Distribution of the sIgE levels in the sensitized population. **(B)** Distribution of the allergens counts in the sensitized population. Single: 1 allergen; Some: 2 to 4 allergens; Multiple: 5 or more allergens. CU, chronic urticaria; AD, atopic dermatitis; AR, allergic rhinitis; AS, asthma. *Bonferroni correction was used for *post-hoc* analysis. Compared with the CU group, P<0.0167 (0.05/3) was considered statistically significant.

**Figure 2:**
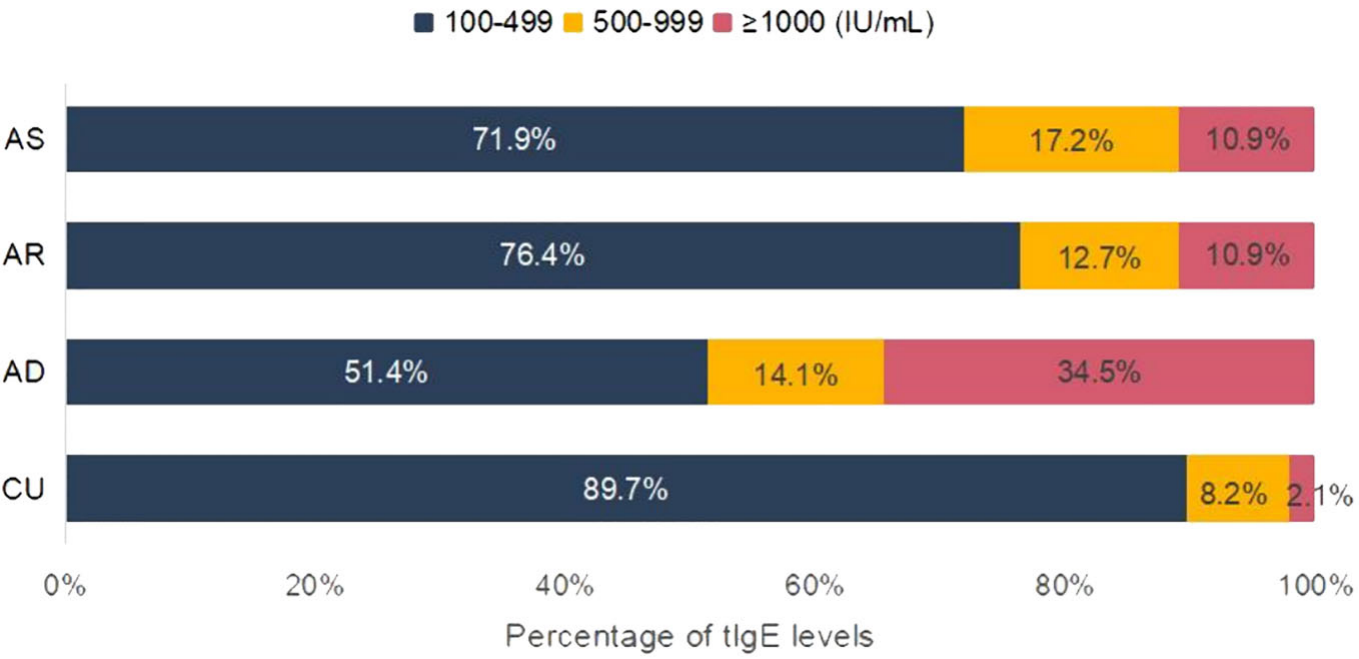
Analysis of tIgE elevation characteristics. Included were patients with elevated tIgE. CU, chronic urticaria; AD, atopic dermatitis; AR, allergic rhinitis; AS, asthma.

The distribution of the sIgE levels, the positive allergen counts, the allergen categories, and tIgE levels among different diseases were compared. For sIgE levels, the positive sIgE in CU was mainly distributed in low level (grade 1 to 2), accounting for 62.6%, and only 6.9% in high level (grade 5 to 6), which was significantly different from AD and AR, but there was no statistical difference with AS (**Figure 1A**). Regarding the number of positive sIgE detected, nearly half of the CU cases only show sensitization to a single allergen, which was also significantly different from AD and AR, but there was no statistical difference with AS (**Figure 1B**). For the allergen categories, the top three allergens in CU patients are dust mites, insects, and animal-derived food allergens, in AD they were dust mites, animal dander, and animal-derived food allergens, in AS they were dust mites, pollen, and inhalant fungal allergens, and in AR they were dust mites, pollen, and animal-derived food allergens. The proportion of CU patients sensitized to animal dander allergens (9.9%) was significantly lower than that among AD patients (21.6%); similarly, the proportion of CU patients sensitized to fungal allergens (6.9%) was significantly lower than that among AS patients (20.9%) (**Table 4**). Regarding the tIgE levels, 89.7% of CU patients had slightly elevated tIgE (100-499 IU/mL), the proportion significantly higher than that of the three classic allergic diseases. In contrast, only 2.1% of CU patients had highly elevated tIgE (≥1000 IU/mL), which was significantly lower than that of the three classic allergic diseases (**Figure 2**) (**Supplementary Table S2**).

**Table 4:** Distribution of allergen categories in sensitized populations^a^.

n (%)	CU (n=131)	AD (n=213)	AR (n=65)	AS (n=67)	*P* ^b^
Dust mites	93 (71.0)	159 (74.6)	56 (86.2)	47 (70.1)	0.103
Pets	13 (9.9)	46 (21.6)*	9 (13.8)	7 (10.4)	0.015
Insects	21 (16.0)	19 (8.9)	4 (6.2)	7 (10.4)	0.111
Pollens	18 (13.7)	45 (21.1)	12 (18.5)	16 (23.9)	0.261
Airborne fungus	9 (6.9)	17 (8.0)	8 (12.3)	14 (20.9)*	0.009
Animal-derived food allergens	21 (16.0)	54 (25.4)	12 (18.5)	3 (4.5)	0.001
Plant-derived food allergens	6 (4.6)	14 (6.6)	2 (3.1)	7 (10.4)	0.308

IQR, interquartile range; CU, chronic urticaria; AD, atopic dermatitis; AR, allergic rhinitis; AS, asthma.

a Included were patients with positive sIgE.

b Pearson chi-squared test.

*Bonferroni correction was used for *post-hoc* analysis. Compared with the CU group, P<0.0167 (0.05/3) was considered statistically significant.

### 3.6 CU was more prone to the inconsistency between sIgE and tIgE results

The concordance between sIgE and tIgE results was also one of the characteristics of IgE sensitization. The concordance rate of sIgE and tIgE results in CU patients was only 65.4%, lower than that in AD (72.0%), AS (73.4%), AR (76.2%), and HC (71.6%), but there was no statistical difference among the groups. In terms of the specific distribution of discordant results, the proportion of CU patients who were sIgE (+) tIgE (+) was 23.7%, the lowest among the four diseases, significantly lower than in AD and AR patients, but not statistically different from AS. Correspondingly, the proportion of CU patients who were sIgE (-) tIgE (-) was the highest at 41.7%, significantly higher than that in AD, but with no statistical difference from AR and AS. Additionally, the proportion of CU patients who were sIgE (-) tIgE (+) (25.3%) was significantly higher than in all classic allergic diseases (**Table 5**).

**Table 5:** Distribution characteristics of sIgE and tIgE in different populations.

	CU	AD	AR	AS	*P* ^a^
Group1 sIgE (+) tIgE (+)	94 (23.7)	159 (38.7)*	48 (47.5)*	47 (33.8)	<0.001
Group2 sIgE (+) tIgE (-)	37 (9.3)	54 (13.1)	17 (16.8)	20 (14.4)	0.112
Group3 sIgE (-) tIgE (+)	100 (25.3)	61 (14.8)*	7 (6.9)*	17 (12.2)*	<0.001
Group4 sIgE (-) tIgE (-)	165 (41.7)	137 (33.3)	29 (28.7)*	55 (39.6)	0.024

CU, chronic urticaria; AD, atopic dermatitis; AR, allergic rhinitis; AS, asthma.

a Pearson chi-squared test.

*Bonferroni correction was used for *post-hoc* analysis. Compared with the CU group, P<0.0167 (0.05/3) was considered statistically significant.

## 4 Discussion

IgE sensitization represents a pivotal component in the evolution of atopy and the genesis of allergic disorders (22). The nuances of IgE sensitization offer us critical insights that are invaluable for elucidating patients’ atopic profiles, investigating the etiology of diseases, and managing their conditions effectively (23). Despite its significance, a limited number of studies have thoroughly explored the characteristics of IgE sensitization in CU. Our study embarked on a comprehensive examination of the distribution patterns of allergen-sIgE and tIgE in CU, conducting a meticulous comparison with their counterparts in established allergic diseases, such as AD, AR, and AS. By doing so, it generated a wealth of data that sheds light on the disparities in IgE sensitization profiles between CU and classical allergic diseases. Furthermore, our study offers valuable clues suggesting the potential existence of a subtype of CU that is intimately associated with atopy.

Detection of allergen-sIgE in serum and allergen skin prick tests (SPT) are the most frequently used methods for identifying sensitization to specific allergens. In our study, through serum sIgE detection, we established that the sensitization rate among CU patients was 33.1%, significantly elevated compared to the general population. This underscores the critical role of sIgE sensitization as a characteristic of CU patients. It is noteworthy that 12.4% of the CU patients included in our study had documented histories of other comorbid atopic diseases, within this subgroup, a significantly higher proportion tested positive for sIgE, with a positivity rate of 66.3% (data not shown). While the inclusion of these patients may influence the overall sIgE positivity rate, it is a reflection of the clinical reality where a subset of CU patients have concurrent atopic conditions. Literature indicate that sIgE sensitization rates in CU patients can range from 17.2% to 95.83% (6, 9, 24, 25), with variations potentially attributable to the detection methodologies employed across studies, as well as ethnic and geographic disparities among patient cohorts.

Our research revealed a gender-based disparity in allergen positivity rates among CU patients, with male patients with CU showing a higher rate of sensitization to allergens. As far back as two decades ago, Kulthanan et al. discovered through SPT that sensitization to house dust mites (HDM) was more prevalent among male CU patients than their female counterparts (26). Similarly, Ping et al. confirmed through sIgE detection that males exhibit a heightened susceptibility to allergen sensitization in urticaria patients (27). While these studies and our study have indicated a potential gender-based disparity in the prevalence of allergen sensitization in CU, the biological underpinnings of this gender discrepancy are complex and need further study. We speculate it likely involve a combination of genetic, environmental, and immunological factors. For instance, it has been suggested that sex hormones may influence the immune response, with potential effects on IgE production and allergen sensitization Additionally, differences in environmental exposures, such as occupational settings, could contribute to the variances seen between males and females (28, 29). It is also noteworthy that children have been observed to be more prone to sensitization reactions, a finding that might be attributed to their immature digestive and immune systems (30). In our research findings, it is indeed demonstrated that the positive rate of sIgE in the minors group is significantly higher than that in the adults group for classic allergic diseases such as AD, AR, and AS. However, this difference is not significant in CU. The reason behind this phenomenon is not yet clear, but it also indirectly suggests the differences in IgE sensitization characteristics between CU and classic atopic diseases, as well as the distinct pathophysiological mechanisms underlying CU. Further investigation with a larger sample size and more detailed age stratification is needed to clarify the relationship between age and allergen sensitization in CU patients.

Elevated levels of tIgE are a common finding in patients with CU and are intimately linked to the clinical manifestations of the disease (31). An increased tIgE is suggestive of heightened disease activity, prolonged illness duration, a favorable response to omalizumab therapy, a swift relapse post-medication cessation, and a diminished response to cyclosporine treatment (31–33). Extensive research utilizing diverse detection methodologies and varying thresholds has reported an increase in tIgE levels in 18% to 82% of cases, with the majority of studies affirming that over 50% of CU patients exhibit elevated tIgE levels (33). The median or mean tIgE levels reported in the literature are between 100 to 300 IU/ml (34). In our study, employing a benchmark threshold of 100 IU/ml, we identified that 49% of CU patients had elevated tIgE levels, with a median tIgE level (IQR) of 96.7 (43.1, 193.0) IU/ml. This finding aligns with previous studies, underscoring the prevalence of elevated tIgE levels among CU patients. In a departure from prior studies, our research conducted a direct comparison of tIgE levels and their distribution between CU and classical allergic diseases, such as AD, AR and AS. We discovered that CU patients do not show a significantly lower proportion or overall level of elevated tIgE compared to those with classical allergic diseases, with AD being the only exception, exhibiting notably higher tIgE levels. However, an examination of the distribution of patients with varying degrees of elevated tIgE levels reveals a key distinction: a vast majority (89.7%) of CU patients have tIgE levels in the slightly elevated range and a stark minority of CU patients have tIgE levels in the highly elevated range. Both of these proportions are significantly different from those observed in other classic atopic diseases. For instance, over 30% of patients with atopic dermatitis have tIgE levels surpassing 1000 IU/mL, contrasting with only 2.1% of CU patients exceeding this threshold. In the context of atopic diseases, tIgE levels are indicative of the degree of IgE sensitization and the severity of atopy in affected individuals. Our findings suggest that, while a significant proportion of patients with CU may possess an underlying atopic predisposition, the magnitude of IgE sensitization and the intensity of atopy in these patients are comparatively reduced relative to those with classical atopic diseases. The underlying biological mechanisms responsible for this observation remain to be elucidated; however, they may be correlated with the impaired skin barrier function characteristic of AD, which could conceivably lead to an exacerbation of IgE production (35, 36). Further research is warranted to dissect the intricate immunological pathways that underpin these differences.

The level of allergen sIgE is a direct indicator of the severity of a patient’s allergic response to a particular allergen (37). The variety of allergens to which a patient is sensitized offers insights into the extent of their allergic profile, indirectly reflecting the overall degree of IgE sensitization (38). Historically, there has been a dearth of research focused on sIgE sensitization levels and the spectrum of allergens in CU. Our study pioneers a comprehensive comparison between CU and classical allergic diseases, such as AD and AR, in terms of sIgE sensitization levels and the count of sensitizing allergens. Notably, we observed that CU patients are more likely to exhibit sensitization at lower levels (grades 1 to 2) and to a single allergen, as opposed to the patterns seen in AD and AR. It comes as no surprise that CU tends to display a less pronounced sensitization profile compared to classical allergic diseases, a finding that indirectly mirrors ongoing clinical debates about the nexus between CU and allergic conditions. Impaired cutaneous or mucosal barriers are more readily penetrated by exogenous allergens and environmental irritants, thereby predisposing individuals to the activation of type 2 inflammation, which is associated with heightened IgE synthesis and augmented IgE-mediated sensitization (39). Furthermore, IgE is known to undergo class switching not only in lymph nodes and the spleen but also within mucosal tissues (40, 41). CU typically presents as hives on the skin, sometimes accompanied by angioedema, without overt mucosal damage or impairment of the skin barrier. This clinical presentation helps explain the observed differences in sensitization levels and allergen breadth between CU and classical allergic diseases. However, it does not negate the tangible presence of allergen sensitization in CU. It is also important to note that this study did not isolate CU patients with atopic backgrounds for a comparative analysis with classical allergic diseases. CU encompasses a variety of endogenous subtypes, and subtypes such as the autoimmune chronic spontaneous urticaria (CSU) type IIb (42, 43), which does not involve IgE sensitization, contribute to the divergence in sIgE sensitization characteristics when comparing CU as a whole to classical allergic diseases.

In clinical practice, allergen-sIgE and tIgE are often used in conjunction to assess a patient’s atopic background (44). However, discrepancies between these two measures are not rare (45). Previous studies have yet to delve into the variances in the discordance between sIgE and tIgE, particularly when comparing CU to other classic allergic conditions. Our findings reveal a higher incidence of incongruence between sIgE and tIgE results in CU as compared to classical allergic diseases. This is exemplified by a notably increased proportion of patients exhibiting negative sIgE yet presenting with elevated tIgE levels. This suggests a heightened variability in tIgE levels within CU. Elevated tIgE levels are indeed indicative of an atopic background, yet they can be influenced by a myriad of factors, including parasitic infections, malignancies, and overall immune status (46). The alignment—or lack thereof—between sIgE and tIgE in CU indirectly corroborates the notion that CU patients possess a more intricate immune profile compared to those with classical allergic diseases. Assessing the atopic background in CU requires a comprehensive evaluation that integrates both sIgE and tIgE results, acknowledging the inherent propensity for discordance between these markers in CU.

There are several limitations to this study that warrant acknowledgment. Firstly, it is retrospective in nature. In the retrospective data, the clinical diagnoses of the patients did not include a precise classification of CU, hence this study was unable to conduct a specific subtype analysis of CU. Consequently, the study did not fully demonstrate the heterogeneity of CU and the potential influence of various subtypes on IgE sensitization. Secondly, the sample size is relatively modest, which may introduce biases that can only be mitigated by future large-scale, prospective studies. The sIgE testing in this study was confined to serological assessments and did not include confirmatory skin prick tests, a limitation that means serum sIgE levels alone might not accurately represent the full spectrum of a patient’s sensitization profile. Thirdly, the categorizing continuous variables into classes in our study may introduce some loss of precision in our results, but it did not significantly impact the overall conclusions of this study. Furthermore, the absence of comprehensive clinical data precluded a detailed correlation analysis between the clinical manifestations of CU and IgE sensitization features. Bridging this gap will be a priority in the design of future, forward-looking research endeavors.

## 5 Conclusion

While traditional perspectives have not classified CU as an allergic disease, emerging research indicates a significant atopic phenomenon within the CU patient population compared to the general population. A subset of individuals with CU demonstrate notably higher rates of allergen-sIgE positivity and elevated levels of tIgE when compared to healthy individuals, which underscores the objective presence of atopy within this subset of CU patients. The debate over whether CU should be considered an atopic disease continues, and the characteristics of IgE sensitization in CU may hold the key to resolving this controversy. Our study suggests that the IgE sensitization profile in CU differs from that of well-established allergic diseases like AD, AR, and AS. CU patients are more likely to show sensitization to a single allergen, have lower levels of sensitization, and display slightly elevated tIgE levels. Furthermore, the agreement between sIgE and tIgE results is less consistent in CU. These findings indicate that CU exhibits certain differences in atopy compared to classic allergic diseases, and future research should focus on in-depth studies of the clinical subtype of atopic CU.

## Data Availability

The raw data supporting the conclusions of this article will be made available by the authors, without undue reservation.
