# Supplementary figures and images for: Distinct IgE sensitization profiles in chronic urticaria: a comparative study with classic allergic diseases
Source: Front Immunol. 2024 Dec 5;15:1458839. doi: 10.3389/fimmu.2024.1458839 (PMC11655319; doi:10.3389/fimmu.2024.1458839)

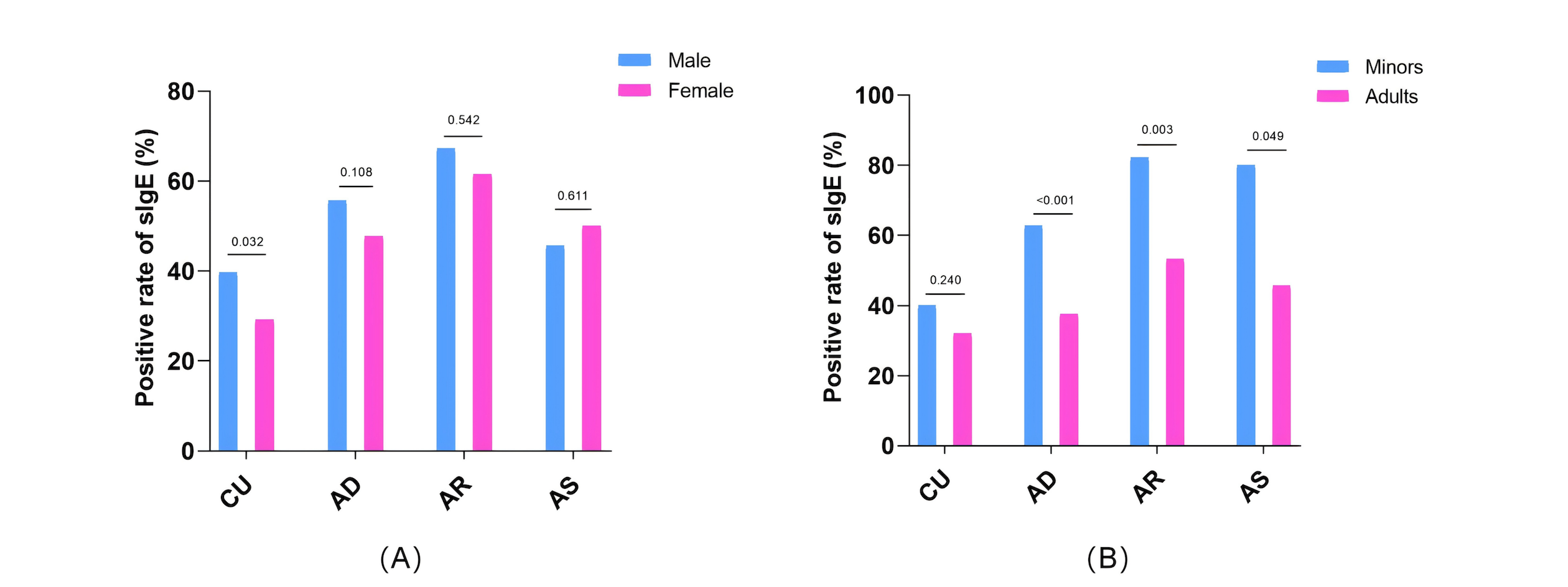

Supplement: Supplementary file 1 [file Image1.tif]
