# Supplementary material for: Distinct IgE sensitization profiles in chronic urticaria: a comparative study with classic allergic diseases
Source: Front Immunol. 2024 Dec 5;15:1458839. doi: 10.3389/fimmu.2024.1458839 (PMC11655319; doi:10.3389/fimmu.2024.1458839)
Supplement: Supplementary file 2 [file Table1.docx]

**Supplementary Table 1** Subgroups of allergens

| Subgroups | Allergens |
| --- | --- |
| Dust mites | *Dermatophagoides pteronyssinus* (*D. pteronyssinus*), *D. farinae*, *Blomia tropicalis* |
| Pets | Cat dander, Dog dander |
| Insects | Cockroach, Silk |
| Pollens | Dwarf ragweed, Artemisia, Humulus, Quinoa/amaranthus, Juniper/birch, Platanus/ash, Alder/poplar /willow/beech/oak/walnut, June grass/ryegrass/timothy, Maple/mulberry/acacia/elm/cypress/paper mulberry |
| Airborne fungus | Aspergillus fumigatus, Candida/Penicillium/Mycosporium/Alternaria /Aspergillus niger |
| Animal-derived food allergens | Egg yolk, Egg white, Milk, Beef/lamb, Fish, Shrimp/crab |
| Plant-derived food allergens | Peanut/soybean, Sesame, Wheat/buckwheat, Cashew/pistachio/hazelnut/almond/walnut, Peach/apple/mango/lychee/strawberry |

**Supplementary Table 2** Analysis of tIgE elevation characteristics^a^

| tIgE, IU/mL | CU  (n=194) | AD  (n=220) | AR  (n=55) | AS  (n=64) | *P* | | | |
| --- | --- | --- | --- | --- | --- | --- | --- | --- |
|  |  |  |  |  | Overall^b^ | CU vs. AD^c^ | CU vs. AR^c^ | CU vs. AA^c^ |
| 100-499 | 174 (89.7) | 113 (51.4) | 42 (76.4) | 46 (71.9) | <0.001 | <0.001 | 0.010 | <0.001 |
| 500-999 | 16  (8.2) | 31 (14.1) | 7  (12.7) | 11 (17.2) | 0.167 | - | - | - |
| ≥1000 | 4  (2.1) | 76 (34.5) | 6  (10.9) | 7  (10.9) | <0.001 | <0.001 | 0.009 | 0.006 |

Abbreviations: CU, chronic urticaria; AD, atopic dermatitis; AR, allergic rhinitis; AS, asthma.

^a^ Included were patients with elevated tIgE.

^b^ Pearson chi-squared test.

^c^ Pearson chi-squared test. Using the Bonferroni correction, *P*<0.0167 (0.05/3) was considered statistically significant.
